# Supplementary material for: Symmetry in category systems across languages
Source: Nat Commun. 2026 Jan 14;17:358. doi: 10.1038/s41467-025-67463-4 (PMC12805004; doi:10.1038/s41467-025-67463-4)
Supplement: Supplementary file 2 — Supplementary Information [file 41467_2025_67463_MOESM2_ESM.pdf]

Supplementary Information

Charles Kemp

Supplementary Discussion

Privileged points

Privileged points play two roles in our theory. First, because these points must be named, they rule out category systems in which privileged points lie on category boundaries. This constraint ensures that symmetric category systems for bisected linear structures have an odd number of categories. Second, in the case of bisected circular structures, privileged points determine the transformations with respect to which a category system must be invariant.

Season terms provide some evidence that privileged points can vary across cultures. If a culture considers the summer and winter solstices as privileged points, then the only symmetric two-category system divides the year into halves that roughly correspond to a hot season and cold season, and the boundaries between these seasons lie at the equinoxes. If a culture considers the equinoxes as privileged points, then a symmetric two-category system divides the year into halves that correspond roughly to a period of rising temperature and a period of falling temperature, and the boundaries between these seasons lie at the solstices. In her work on Native American time reckoning, Cope [3, p 135] notes that in cases where seasonal systems have two seasons, these seasons "may mark the natural periods of cold and heat — as among the Haida, Maidu, Navaho, Bannock, Blackfoot, Arikara, Kiowa, and Choctaw; or the division may be determined by the solstices, as seen among the Bella Coola, Makaw, Juanenfo, Hopi, Zufi, and Hano." This observation suggests that there may be variation across cultures in whether the solstices or the equinoxes are taken as privileged points.

In the case of the bisected circular structure, the identity of the privileged points is not needed in order to make predictions about the parity of category systems. For seasons, our theory predicts that category systems tend to have even sizes regardless of whether the privileged points are the solstices or the equinoxes. Similarly, for cardinal

directional systems, our theory predicts that systems tend to have even sizes regardless of whether the privileged points correspond to east and west or north and south.

Supplementary Methods

Distribution of languages across areas and families

Domain	Africa	Australia	Eurasia	North America	Papunesia	South America	Total
deictic day names	11	0	67	32	39	7	156
tense	35	19	44	49	40	46	233
seasons	4	24	1	21	1	0	51
moon phases	9	1	8	11	10	10	49
locational (cardinal)	0	87	0	0	0	0	87
siblings	105	105	202	140	443	86	1081
parent's siblings	110	99	194	129	409	77	1018
grandparents	61	95	192	128	241	91	808
grandchildren	15	76	144	59	81	44	419
nieces+nephews	60	74	162	107	254	67	724
cousins	86	80	177	110	279	71	803
social categories	0	331	0	0	0	0	331
locational (other)	0	81	0	0	1	0	82
color	17	5	7	26	23	32	110
life forms	17	13	14	47	32	21	144
spatial demonstratives	46	11	84	22	19	11	193

Table S1: Distribution across geographic areas for each data set.

Tables S1 and S2 show distributions across areas and language families for all data sets described in the main text along with the spatial demonstrative data analyzed later in this document. All counts shown represent the number of languages included in the statistical analyses.

Domain	Algic	Atlantic-Congo	Austroasiatic	Austronesian	Bunaban	Great Andamanese	Gunwinyguan	Indo-European	Iwaidjan Proper	Kiowa-Tanoan	Maningrida	Mongolic-Khitan	Nakh-Daghestanian	Nuclear Trans New Guinea	Nyulnyulan	Otomanguean	Pama-Nyungan	Pano-Tacanan	Tangkic	Tupian	Turkic	Uto-Aztecan	Afro-Asiatic	Other	Total
deictic day names	3	4	2	34	0	0	0	30	0	0	0	7	0	3	0	14	0	1	0	0	9	7	4	38	156
tense	1	12	4	11	1	0	2	9	1	1	0	1	4	10	1	4	4	2	1	4	1	7	7	145	233
seasons	3	3	0	1	1	0	2	0	1	3	0	0	0	0	2	0	12	0	0	0	0	3	0	20	51
moon phases	4	5	1	10	0	2	0	0	0	0	0	0	0	0	0	0	1	1	0	2	0	0	2	21	49
locational (cardinal)	0	0	0	0	2	0	3	0	2	0	2	0	0	0	3	0	69	0	1	0	0	0	0	5	87
siblings	26	97	6	308	0	0	2	96	0	1	0	1	24	48	2	0	90	1	3	31	5	31	6	303	1081
parent’s siblings	27	102	7	284	0	0	2	89	0	1	0	1	24	44	2	0	84	1	3	31	5	30	6	275	1018
grandparents	27	56	5	160	0	0	2	92	0	0	0	1	24	39	2	0	80	1	3	31	5	30	2	248	808
grandchildren	1	11	5	40	0	0	2	75	0	0	0	1	19	18	1	0	62	0	3	19	3	11	2	146	419
nieces+nephews	26	53	4	152	0	0	2	84	0	0	0	1	17	36	1	0	60	0	3	26	4	13	4	238	724
cousins	27	79	4	164	0	0	2	87	0	1	0	1	24	41	1	0	67	1	3	28	4	14	4	251	803
locational (other)	0	0	0	1	2	0	5	1	2	0	3	0	0	0	3	0	54	0	3	0	0	0	0	8	82
color	2	12	0	7	0	0	0	9	0	0	0	0	0	4	0	6	3	7	0	1	0	5	1	53	110
life forms	5	13	2	16	0	0	4	4	1	2	0	0	0	11	0	6	7	2	0	2	0	12	0	57	144
spatial demonstratives	2	17	8	10	0	0	0	42	0	0	0	0	1	2	0	1	7	1	0	2	1	4	12	83	193

Table S2: Distribution across language families for each data set. The families shown here were chosen to include the five most common families for each data set.

Kinship data

Table S3 shows the full set of kin types used to define the six subdomains used in the kinship analysis. As described in the Methods, we counted categories rather than terms, and treated all terms for a language that referred to the same set of kin types as a single category. This approach deliberately allows for overlapping categories: for example, Mandarin Chinese includes terms for brother (兄弟) and sister (姐妹) in addition to terms for older brother (哥哥), younger brother (弟弟), older sister (姐姐) and younger sister (妹妹), producing six sibling categories in total.

Subdomain	Kin types
siblings	mB, mZ, meB, myB, meZ, myZ, fB, fZ, feB, fyB, feZ, fyZ
parent's siblings	mMB, mMZ, mFeB, mFyB, mFeZ, mFyZ, mMeZ, mMyZ, mMeB, mMyB, fMB, fMZ, fFeB, fFyB, fFeZ, fFyZ, fMeZ, fMyZ, fMeB, fMyB
grandparents	mFF, mFM, mMF, mMM, fFF, fFM, fMF, fMM
grandchildren	mSS, mSD, mDS, mDD, fSS, fSD, fDS, fDD
nieces+nephews	mBS, mBD, mZS, mZD, meBS, myBS, meBD, myBD, meZS, myZS, meZD, myZD, fBS, fBD, fZS, fZD, feBS, fyBS, feBD, fyBD, feZS, fyZS, feZD, fyZD
cousins	mFZD, mFBD, mBD, mZD, mFBS, mFZS, mMBS, mMZS, mFeBS, mFyBS, mFeZS, mFyZS, mFeBD, mFyBD, mFeZD, mFyZD, mMeBS, mMyBS, mMeZS, mMyZS, mMeBD, mMyBD, mMeZD, mMyZD, mFBeS, mFByS, mFZeS, mFZyS, mFBeD, mFByD, mFZeD, mFZyD, mMBeS, mMByS, mMZeS, mMZyS, mMBeD, mMByD, mMZeD, mMZyD, fFZD, fFBD, fBD, fZD, fFBS, fFZS, fMBS, fMZS, fFeBS, fFyBS, fFeZS, fFyZS, fFeBD, fFyBD, fFeZD, fFyZD, fMeBS, fMyBS, fMeZS, fMyZS, fMeBD, fMyBD, fMeZD, fMyZD, fFBeS, fFByS, fFZeS, fFZyS, fFBeD, fFByD, fFZeD, fFZyD, fMBeS, fMByS, fMZeS, fMZyS, fMBeD, fMByD, fMZeD, fMZyD

Table S3: Kin types included in each kinship subdomain. `m` and `f` indicate a male and female speaker respectively. `B`, `Z`, `F`, `M`, `S`, and `D` indicate brother, sister, father, mother, son and daughter. `e` and `y` indicate elder and younger. For example, `fyZ` is a female speaker's younger sister.

Allowing overlapping categories means that we are as faithful as possible to the underlying data, but our counts for some languages are distorted because of noise and missing entries in the Kinbank data set. For example, the Latvian grandparent data include vecmāmiņa (grandmother) but also vecmāmiņa, which has a different diacritic on the penultimate letter and is only listed for paternal grandmother of a male speaker (`mFM`). Our approach treats these terms as two overlapping categories, which means that the size of the Latvian grandparent system is seven rather than six.

To test whether our results are robust to noise in the underlying data, we ran a second kinship analysis using data compiled by Murdock [5] and released as part of the supporting material accompanying [4]. Murdock's data

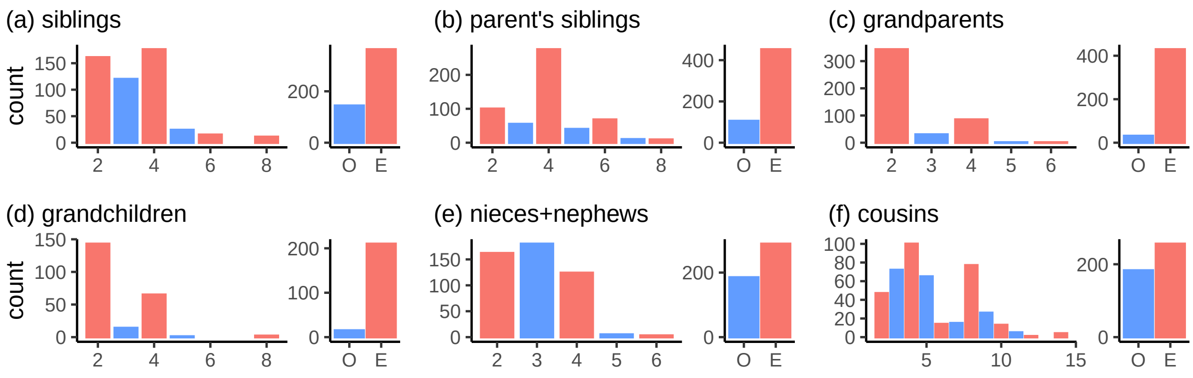

Figure S1: Size distributions based on the kinship data of Murdock [5]. Each panel shows a distribution of system sizes across languages (left), and a summary plot showing the number of odd (O, blue) and even (E, red) systems (right).

are less comprehensive than Kinbank but cleaner, and specify how each subdomain is partitioned into a set of non-overlapping categories.[1] When analyzing the Murdock data, each subdomain is defined using only the most fine-grained kin types listed for each subdomain in Table S3. For example, the eight kin types for sibling include elder brother, younger brother, elder sister and younger sister of a male speaker (`meB`, `myB`, `meZ`, and `myZ`) along with corresponding kin types for a female speaker. For nieces and nephews, the Murdock data capture only terms used by a male speaker. For this domain only, we therefore restricted the set of kin types further to include only kin types relative to a male speaker.

Size distributions based on the Murdock data are shown in Figure S1, and are broadly compatible with the Kinbank results shown in Figure 2. The biggest difference between the two is that the Murdock results show a less pronounced preference for even systems in the case of nieces and nephews. Recall, however, that the Murdock niece and nephew data are unbalanced in the sense that they incorporate only terms for a male speaker, which means that the Kinbank result for nieces and nephews may be more reliable than the Murdock result.

Symmetry and color-naming

Consider a color space like the example in Figure S2a, where white lies at the center and saturated colors lie around the periphery. Suppose that reflections in the red-green and blue-yellow axes are both symmetries of the space. Because the interior of the space is occupied, symmetric partitions may have an odd or an even number

[1] Kinbank can also be used to create systems of non-overlapping categories, and Passmore [7] achieve this by taking the first term listed for each fine-grained kin type.

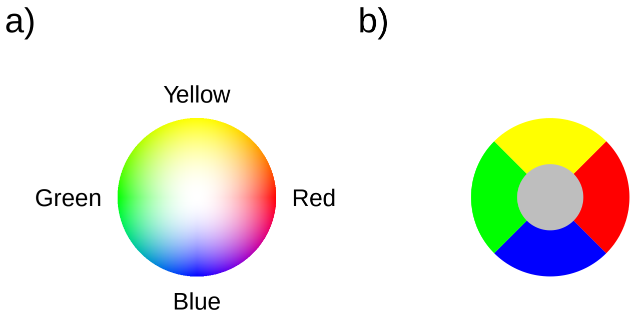

Figure S2: Partitioning a color space into categories. (a) A two-dimensional color space with axes corresponding to red-green and blue-yellow. (b) A symmetric color system of size 5. Flat regions of color show points assigned to the same category.

of categories. Figure S2b shows a symmetric system of size 5, where the category shown in grey includes highly unsaturated colors. If the grey category shrank to a point then disappeared, the resulting partition would be a symmetric system of size 4. A similar conclusion applies to a three-dimensional color solid such as CIELAB color space. Because the interior of the solid is occupied, symmetric partitions of the solid may have an odd or an even number of categories.

Symmetry and spatial demonstrative systems

The main text focuses on the parity of category systems but suggests that symmetry can also be investigated in domains where symmetry makes no strong predictions about parity. We illustrate by considering systems of spatial demonstratives such as *here* and *there* in English. Following Chen et al. [2], we consider deictic-centric systems that refer to positions in space relative to an imagined deictic center. Each system corresponds to a partition of a grid with two dimensions. The first dimension is distance, and the top-left system in Figure S3a shows a system with two distance levels D1 and D2. The second dimension is orientation, and the top-left system in Figure S3a distinguishes between cases in which the referent is at a given distance level (place), moving towards a distance level (goal) or coming from a distance level (source).

Again following Chen et al. [2], we work with a set of spatial demonstrative systems compiled by Nintemann et al. [6]. Chen et al. [2] analyze 220 languages, but systems for 26 of these languages are incomplete in the sense that they do not specify terms for an entire grid. We dropped these 26 languages, and the 194 languages that remained included two (Serbian and Croatian) with the same glottocode. Since the systems for these languages are

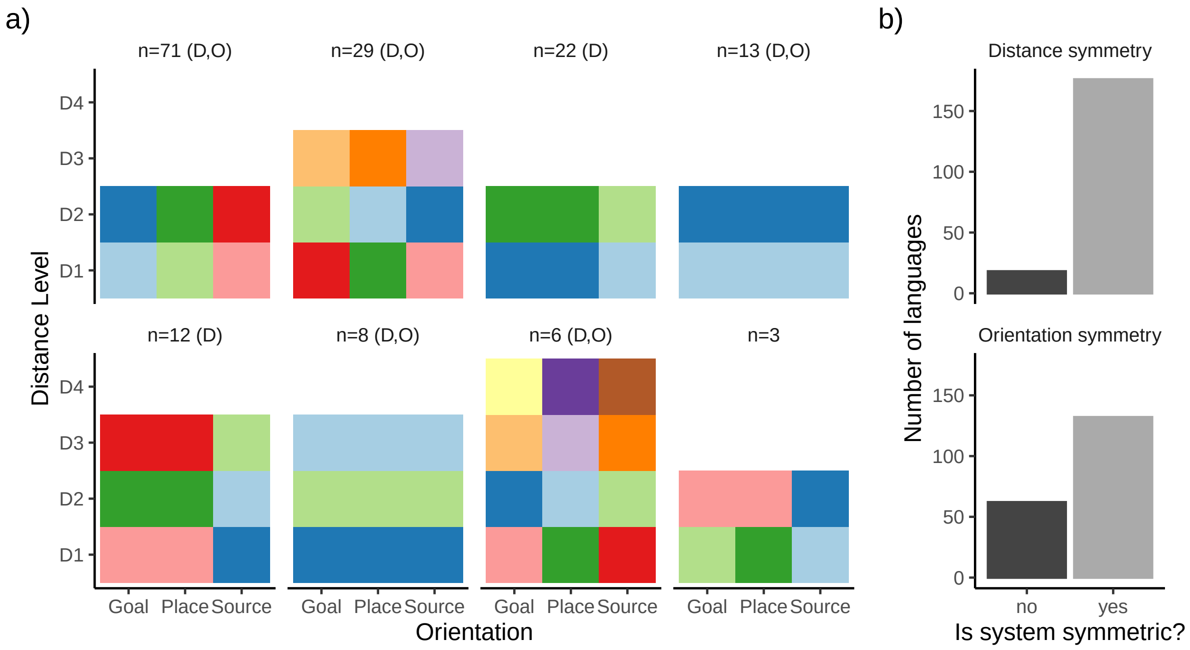

Figure S3: Analysis of spatial demonstrative systems across languages. (a) The eight most common spatial demonstrative systems in the Nintemann et al. [6] data. Panel labels show the frequency of each system, and D and O indicate systems that show distance symmetry and orientation symmetry respectively. (b) Number of languages with spatial demonstrative systems that show distance symmetry (top) and orientation symmetry (bottom).

isomorphic, we dropped one of the two leaving 193 languages for analysis. The eight most common systems are shown in Figure S3a.

The most common system incorporates two distance levels and has different terms for all 6 combinations of an orientation and a distance level. English includes four demonstratives (*here*, *there*, *from here* and *from there*), and is an instance of the third most common system, which distinguishes between two distance levels but uses the same demonstrative (e.g. *here*) for place and goal.

Given these systems, it is natural to consider two kinds of transformations. The first is a transformation that inverts the distance dimension, and we will say that a system is distance symmetric if it is invariant under this transformation. 7 of the 8 systems in Figure S3a are distance symmetric, as indicated by the D in the labels for these systems. For example, starting with the most common system and inverting the distance dimension still leaves a 6 category system that distinguishes between all elements in the grid.

The second kind of transformation permutes the orientations, and we will say that a system is orientation symmetric if it is invariant under all possible orientation transforms. 6 of the 8 systems in Figure S3a are orientation

symmetric, as indicated by the O in the label for these systems. The English system (third-most common in Figure S3a) is not orientation symmetric, because swapping Goal and Source (or Place and Source) produces a system that is not isomorphic to the original.

The first two systems in Figure S3a illustrate that symmetry makes no strong predictions about parity, because systems that are both distance and orientation symmetric may have either an even or an odd number of categories. The same result obtains even if we consider only systems with exactly two distance levels. These systems are represented by a 2 by 3 grid, and partitions of the grid into rows (two categories) or columns (three categories) are both distance and orientation symmetric.

Even though symmetry does not support strong predictions about parity for this domain, we can still examine how often attested systems have distance and orientation symmetry. Figure S3b shows that 91% of systems in the sample are distance symmetric, and 68% are orientation symmetric. Phylogenetic regressions analogous to those described in the main text indicate that the probability that a new system will have distance symmetry is 0.93 [0.87, 0.98], and the probability of having orientation symmetry is 0.72 [0.61, 0.86], where the intervals are 95% credible intervals. These results therefore suggest that spatial demonstrative systems tend to be symmetric.

Nintemann et al. [6] notes that spatial demonstrative systems tend to make the same distinctions at each distance level: for example, if Place and Goal are merged at level D1 (as they are for the third system in Figure S3a) then they tend to be merged at all levels. This property is closely related to our notion of distance symmetry, and is satisfied if and only a system is distance symmetric. Nintemann et al. [6] also suggest that attested systems tend to treat orientations in one of three ways: (i) they distinguish fully between Place, Goal and Source; (ii) they do not distinguish between Place, Goal and Source; or (iii) they merge Place and Goal but treat Source differently. Patterns (i) and (ii) are related to orientation symmetry, and a system that is orientation symmetric is characterized by one of these patterns. The third pattern, however, points to the influence of factors other than symmetry, and suggests that Place and Goal are psychologically more similar than either is to Source.

Symmetry is also related to the notion of *consistency* introduced by Chen et al. [2]. A spatial demonstrative system is consistent if it makes the same distinctions at each distance level and for each orientation. To quantify consistency, Chen et al. [2] use a consistency score that measures the departure from full consistency using a sum of two terms. The first term is the number of distinct patterns across the rows of the grid representing the system. For example, the final grid shown in Figure S3a has two distinct row patterns (the top row merges Place and Goal but the second row does not), and all other systems shown have a single row pattern. The number of distinct row patterns is conceptually related to distance symmetry, and any distance-symmetric system will have a single row

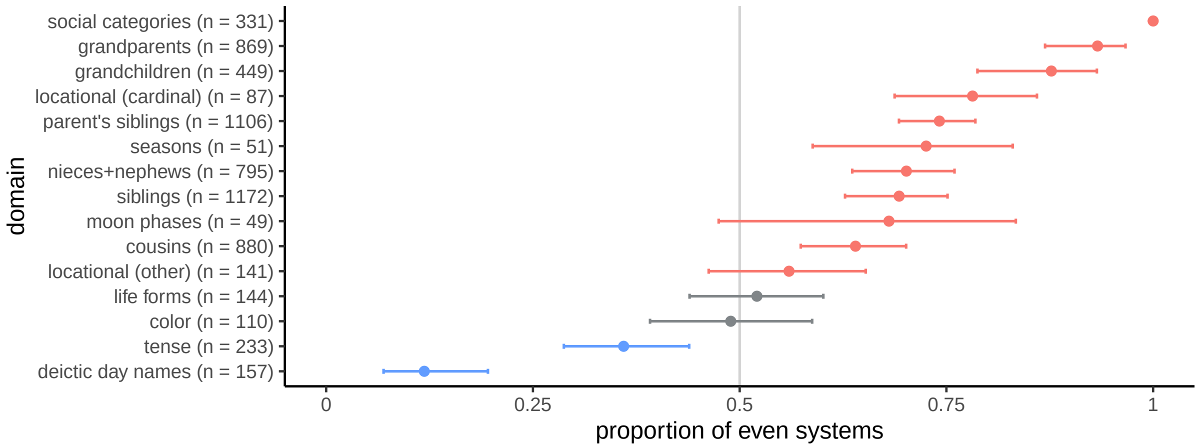

Figure S4: Estimates of the probability that a system is even along with 95% confidence intervals. Each estimate is the intercept of a mixed effects logistic regression model, and a logistic transform has been applied so that an intercept of 0 corresponds to a probability of 0.5. Confidence intervals for domains predicted to be even or odd are shown in red or blue respectively, and confidence intervals for control domains are shown in grey. The values of n along the y-axis show the number of category systems used for each domain.

pattern only. The second term in the consistency score is the number of distinct patterns across the columns of the grid, and all grids in Figure S3a have a single column pattern only. The number of distinct column patterns is conceptually related to orientation symmetry, and an orientation-symmetric system will have a single column pattern only. The third, fifth and eighth grids in Figure S3 show, however, that a single column pattern can also be achieved by systems that lack orientation symmetry.

Our analysis of spatial demonstrative systems illustrates a general approach to testing for symmetry that can be summarized as follows. First, define one or more transformations that apply to the domain under consideration. Second, for each transformation compute the proportion of category systems that are left invariant under the transformation. Testing each transformation separately is useful because attested systems may turn out to be symmetric with respect to some but not all of the transformations considered. For example, Tent [8] considers the symmetry of systems of phonemes, and shows that vowel systems tend to show front-back symmetry — in other words, they tend to be invariant with respect to a transformation that exchanges front vowels and back vowels. Vowel systems, however, are typically not symmetric to a transformation that exchanges high vowels and low vowels.

Frequentist analyses

To check the robustness of the results in Figure 3 we ran a frequentist mixed-effects regression analysis using the `glmmTMB` package [1]. For all domains except the two locational domains we included a random effect for

language family, and the analyses for kinship and non-cardinal directional systems included a random effect for language. 95% confidence intervals for each domain are shown in Figure S4, and are similar to the credibility intervals plotted in Figure 3.

Supplementary References

[1] Brooks, M. E., Kristensen, K., Van Benthem, K. J., Magnusson, A., Berg, C. W., Nielsen, A., Skaug, H. J., Machler, M., and Bolker, B. M. (2017). glmmTMB balances speed and flexibility among packages for zero-inflated generalized linear mixed modeling. *The R journal*, 9(2):378–400.

[2] Chen, S., Futrell, R., and Mahowald, K. (2023). An information-theoretic approach to the typology of spatial demonstratives. *Cognition*, 240:105505.

[3] Cope, L. (1919). *Calendars of the Indians north of Mexico*. University of California Press.

[4] Kemp, C. and Regier, T. (2012). Kinship categories across languages reflect general communicative principles. *Science*, 336:1049–1054.

[5] Murdock, G. P. (1970). Kin term patterns and their distribution. *Ethnology*, 9:165–207.

[6] Nintemann, J., Robbers, M., and Hober, N. (2020). *Here–Hither–Hence and related categories: A cross-linguistic study*. Walter de Gruyter GmbH & Co KG.

[7] Passmore, S. (2023). The global recurrence and variability of kinship terminology structure. https://doi.org/10.31234/osf.io/b7da5.

[8] Tent, J. (1993). Phonetic symmetry in sound systems. *Symmetry: Culture and Science*, 4(4):345–368.
