# Supplementary material for: Symmetry in category systems across languages
Source: Nat Commun. 2026 Jan 14;17:358. doi: 10.1038/s41467-025-67463-4 (PMC12805004; doi:10.1038/s41467-025-67463-4)
Supplement: Supplementary file 3 — Reporting Summary [file 41467_2025_67463_MOESM3_ESM.pdf]

nature portfolio

Corresponding author(s): Charles Kemp

Last updated by author(s): Nov 9, 2025

Reporting Summary

Nature Portfolio wishes to improve the reproducibility of the work that we publish. This form provides structure for consistency and transparency in reporting. For further information on Nature Portfolio policies, see our Editorial Policies and the Editorial Policy Checklist.

Statistics

For all statistical analyses, confirm that the following items are present in the figure legend, table legend, main text, or Methods section.

n/a	Confirmed	
☐	☒	The exact sample size (*n*) for each experimental group/condition, given as a discrete number and unit of measurement
☐	☒	A statement on whether measurements were taken from distinct samples or whether the same sample was measured repeatedly
☒	☐	The statistical test(s) used AND whether they are one- or two-sided<br>*Only common tests should be described solely by name; describe more complex techniques in the Methods section.*
☐	☒	A description of all covariates tested
☒	☐	A description of any assumptions or corrections, such as tests of normality and adjustment for multiple comparisons
☐	☒	A full description of the statistical parameters including central tendency (e.g. means) or other basic estimates (e.g. regression coefficient) AND variation (e.g. standard deviation) or associated estimates of uncertainty (e.g. confidence intervals)
☒	☐	For null hypothesis testing, the test statistic (e.g. *F*, *t*, *r*) with confidence intervals, effect sizes, degrees of freedom and *P* value noted<br>*Give P values as exact values whenever suitable.*
☐	☒	For Bayesian analysis, information on the choice of priors and Markov chain Monte Carlo settings
☒	☐	For hierarchical and complex designs, identification of the appropriate level for tests and full reporting of outcomes
☒	☐	Estimates of effect sizes (e.g. Cohen's *d*, Pearson's *r*), indicating how they were calculated

Our web collection on statistics for biologists contains articles on many of the points above.

Software and code

Policy information about availability of computer code

Data collection	Custom web-scraping code was used to compile some of the data. This code is included in the project repository, which is available at https://github.com/cskemp/parity and archived at https://doi.org/10.5281/zenodo.17429647.
Data analysis	Custom code was used for the analyses, and this code is included in the project repository. The statistical analyses used version 2.23.0 of the brms package.

For manuscripts utilizing custom algorithms or software that are central to the research but not yet described in published literature, software must be made available to editors and reviewers. We strongly encourage code deposition in a community repository (e.g. GitHub). See the Nature Portfolio guidelines for submitting code & software for further information.

Data

Policy information about availability of data

All manuscripts must include a data availability statement. This statement should provide the following information, where applicable:

- Accession codes, unique identifiers, or web links for publicly available datasets
- A description of any restrictions on data availability
- For clinical datasets or third party data, please ensure that the statement adheres to our policy

Data are available at https://github.com/cskemp/parity and archived at https://doi.org/10.5281/zenodo.17429647. Data for day naming, tense, and life forms were

transcribed from previous publications by Tent, Velupillai and Brown. Data for seasons and moon phases are released here for the first time. Data for locational systems were compiled by Hoffmann, Palmer and Gaby and generously provided by these authors. Kinbank data were downloaded from https://github.com/kinbank/kinbank, and the Murdock kinship data analyzed in the supplementary material were downloaded from http://charleskemp.com/kinship/murdockdata.zip. Color data were derived from the wcsR package. Data for Australian social classification were derived from the AustKin website, and the spatial demonstrative data analyzed in the supplementary information were downloaded from https://github.com/cshnican/spatial_demonstratives.

Research involving human participants, their data, or biological material

Policy information about studies with human participants or human data. See also policy information about sex, gender (identity/presentation), and sexual orientation and race, ethnicity and racism.

Reporting on sex and gender	NA
Reporting on race, ethnicity, or other socially relevant groupings	NA
Population characteristics	NA
Recruitment	NA
Ethics oversight	NA

Note that full information on the approval of the study protocol must also be provided in the manuscript.

Field-specific reporting

Please select the one below that is the best fit for your research. If you are not sure, read the appropriate sections before making your selection.

☐ Life sciences ☒ Behavioural & social sciences ☐ Ecological, evolutionary & environmental sciences

For a reference copy of the document with all sections, see nature.com/documents/nr-reporting-summary-flat.pdf

Behavioural & social sciences study design

All studies must disclose on these points even when the disclosure is negative.

Study description	Quantitative
Research sample	The study used existing linguistic data sets and a new data set of lunar phase systems compiled for this project.
Sampling strategy	I compiled the lunar phase data by going through all paragraphs in the Human Relations Area Files that were classified with the subjects “ordering of time” or “ethnometeorology” and that included either “phase” or “phases” and “lunar” or “moon.” I added to the resulting data set by searching Google, Google Scholar and Google Books for combinations of terms and phrases such as “terms for”, “phases of” and “moon.”
Data collection	Data were compiled using an online spreadsheet.
Timing	The lunar phase data were compiled over the second half of 2024.
Data exclusions	Exclusions are documented in the manuscript (e.g. Natleba was excluded from the day-naming dataset because a glottocode could not be determined for this language)
Non-participation	No participants were involved
Randomization	No experimental groups were used

Reporting for specific materials, systems and methods

We require information from authors about some types of materials, experimental systems and methods used in many studies. Here, indicate whether each material, system or method listed is relevant to your study. If you are not sure if a list item applies to your research, read the appropriate section before selecting a response.

Materials & experimental systems

n/a	Involved in the study
☒	☐ Antibodies
☒	☐ Eukaryotic cell lines
☒	☐ Palaeontology and archaeology
☒	☐ Animals and other organisms
☒	☐ Clinical data
☒	☐ Dual use research of concern
☒	☐ Plants

Methods

n/a	Involved in the study
☒	☐ ChIP-seq
☒	☐ Flow cytometry
☒	☐ MRI-based neuroimaging

Plants

Seed stocks	NA
Novel plant genotypes	NA
Authentication	NA
